# Public health considerations for transitioning beyond the acute phase of the COVID-19 pandemic in the EU/EEA

**DOI:** 10.2807/1560-7917.ES.2022.27.17.2200155

**Published:** 2022-04-28

**Authors:** Jonathan E Suk, Anastasia Pharris, Julien Beauté, Edoardo Colzani, Howard Needham, John Kinsman, Rene Niehus, Rok Grah, Ajibola Omokanye, Diamantis Plachouras, Agoritsa Baka, Bastian Prasse, Frank Sandmann, Ettore Severi, Erik Alm, Emma Wiltshire, Bruno Ciancio

**Affiliations:** 1 European Centre for Disease Prevention and Control (ECDC), Stockholm, Sweden

**Keywords:** COVID-19, SARS-CoV-2, pandemic preparedness, global health governance, epidemiology

## Abstract

Many countries, including some within the EU/EEA, are in the process of transitioning from the acute pandemic phase. During this transition, it is crucial that countries’ strategies and activities remain guided by clear COVID-19 control objectives, which increasingly will focus on preventing and managing severe outcomes. Therefore, attention must be given to the groups that are particularly vulnerable to severe outcomes of SARS-CoV-2 infection, including individuals in congregate and healthcare settings. In this phase of pandemic management, a strong focus must remain on transitioning testing approaches and systems for targeted surveillance of COVID-19, capitalising on and strengthening existing systems for respiratory virus surveillance. Furthermore, it will be crucial to focus on lessons learned from the pandemic to enhance preparedness and to enact robust systems for the preparedness, detection, rapid investigation and assessment of new and emerging SARS-CoV-2 variants. Filling existing knowledge gaps, including behavioural insights, can help guide the response to future resurgences of SARS-CoV-2 and/or the emergence of other pandemics. Finally, ‘vaccine agility’ will be needed to respond to changes in people’s behaviours, changes in the virus, and changes in population immunity, all the while addressing issues of global health equity.

## Background

The emergence and rapid global transmission of the severe acute respiratory syndrome coronavirus-2 (SARS-CoV-2) Omicron variant of concern (VOC) serves as both a reason for cautious optimism and a cautionary tale. Given that the attack rate of the Omicron VOC in the population has been very high, that 70% of the European Union/European Economic Area (EU/EEA) population has completed its primary vaccination course, and that the uptake of booster doses has been generally high, it is expected that at the end of the ongoing Omicron wave the vast majority of the EU/EEA population will have built a certain degree of immunological memory against SARS-CoV-2. This could result in a reduced severity of coronavirus disease (COVID-19) in the population going forward. Such a situation would enable countries to more efficiently manage the pandemic [1]. However, it is generally expected that SARS-CoV-2 will become endemic [2], and this may involve repeated reinfections, some degree of seasonality in temperate regions, and/or unanticipated upsurges in cases. For example, following what appeared to be the end of a SARS-CoV-2 Omicron wave and following the accompanying relaxation of measures in many countries, COVID-19 case notification rates started to increase again in 14 of 30 EU/EEA countries by 18 March 2022, impacting hospitalisation and death rates in some of these settings [3]. Following a few weeks of an increase, most countries are now observing declining rates again as of the week ending 17 April 2022, with hospitalisation and death rates similar to pre-Omicron levels. Meanwhile, high levels of global SARS-CoV-2 circulation leads to an elevated risk of viral mutation, and it should not be assumed that each subsequent SARS-CoV-2 variant will lead to less severe disease than its predecessor [4].

The extensive range of non-pharmaceutical interventions (NPIs) implemented in the EU/EEA over the past 2 years has contributed to reductions in COVID-19-related hospitalisations and mortality, particularly before vaccination uptake reached very high levels [5]. However, NPIs have also exacted heavy societal and economic costs. Decisionmakers will need to continue to weigh the effectiveness and societal impact of COVID-19 control measures against the observed severity of COVID-19 at the population level, and information around key variables, including COVID-19 incidence and severity, will continue to vary across time and space. Furthermore, as widely observed up to this point in the COVID-19 pandemic, decisions regarding COVID-19 control measures also occur in political spheres [6], where factors beyond public health evidence often influence decision making [7].

## Towards a pandemic transition phase

Relaxing NPI mitigation measures while SARS-CoV-2 continues to transmit globally implies a shift in focus, from limiting both disease spread and severe outcomes to primarily focusing on managing severe outcomes. It is instructive to view the current context as the beginning of a pandemic transition phase, which cannot be time-stamped but may last for 1–2 years. During this time, healthcare systems will seek to recover from the pandemic strain and make strategic decisions to adapt public health preparedness, response, and surveillance systems capable of managing COVID-19 and other future pandemics over the long term. During this pandemic transition phase, many public health issues will need to be considered, the most important being that countries focus on enhancing pandemic preparedness and response, adapting testing and surveillance strategies, protecting vulnerable groups, expanding capabilities for conducting behavioural research, ensuring ‘vaccine agility’, strengthening global health, and addressing critical knowledge gaps.

## Enhancing pandemic preparedness and response

The COVID-19 pandemic has had many long-ranging impacts on public health as well as on the larger global economic and social sphere in which public health operates. It is imperative that innovations and good practices that emerged during the pandemic are safeguarded. Structured after-action reviews and lessons learned exercises, with sufficient high-level buy-in to ensure that lessons are acted on, should be an important activity area during the pandemic transition phase [8].

A key short- and long-term objective for pandemic preparedness will be to ensure the rapid identification, investigation and assessment of key epidemiological parameters for each new variant of interest or of concern. Arrangements to ensure the timely information sharing from scientific or public health communities on new variants are vital to ensure a globally coordinated response to the pandemic. Operational study protocols, where not already in place, must be developed and implemented so that secondary attack and viral growth rates for newly circulating variants can be assessed and neutralisation studies performed.

As stringent NPIs have many collateral socioeconomic impacts, several population-level NPIs may principally be viewed as a last resort, to be implemented only during high-impact outbreaks. However, it will be important to maintain the possibility to implement the most effective, cost-effective, and socially tolerable measures when needed. Thus, when NPIs are implemented, they should be accompanied by assessments of their effectiveness, cost-effectiveness and public acceptance.

In anticipation of future outbreaks, national preparedness and response plans should be revised and tested routinely, and protocols for quickly assessing the transmissibility and severity of new variants should be put in place. Networks of teams that can be quickly deployed to outbreak situations (whether for respiratory or other types of disease) should be expanded and reinforced, as is envisioned by establishment of the EU Health Task Force [9], to provide more coordinated and operational responses to outbreak settings across Europe.

## Adapting testing strategies and surveillance

Due to reduced severity associated with the Omicron variant and its higher transmissibility making isolation and quarantine less practical, countries are contemplating a shift in testing approach away from diagnosis and case detection and towards routine indicator-based surveillance [10]. Until recently, in most countries, COVID-19 surveillance systems relied on comprehensive collection of testing data. Testing rates (10,000 tests per 100,000 population for week 2022–02 at EU/EEA level) may not be sustainable and their cost-effectiveness is unclear. However, changing testing strategies impact testing indicators and may distort surveillance data in unpredictable ways [11].

ECDC published a COVID-19 surveillance guidance encouraging countries to transition from emergency surveillance for COVID-19 to more sustainable, objective-driven surveillance systems [12]. These systems should allow for integrated surveillance of COVID-19, influenza and other respiratory pathogens that are likely to co-circulate in the population. Such a transition will require adjusting and enhancing existing sentinel schemes used for seasonal influenza surveillance and implementing SARI (severe acute respiratory infection) surveillance.

The contact patterns between individuals are changing over time. The paths of SARS-CoV-2 transmission in the population are determined by the heterogeneous and time-varying physical contacts between individuals, ranging from superspreading events by individuals with many contacts and/or particularly high viral shedding to isolated individuals with only few contacts [13]. Accurate data on temporal contact patterns between individuals for different regions, vaccination status and age will be important for predicting the future course of COVID-19.

The focus of diagnosis testing will increasingly shift to the timely testing of symptomatic people with underlying risk factors for severe COVID-19 who may benefit from early antiviral treatment and the testing of people who have contact with vulnerable populations such as healthcare workers in acute and long-term care settings. Since groups that are likely to have mild disease (i.e., young and healthy individuals) are also those more likely to be the first infected in an upsurge, it is important to keep a representative sample of these groups to be tested for surveillance purposes. Screening testing (i.e., testing individuals without symptoms) should be restricted to high-risk settings. Although the cost-effectiveness of community screening to prevent future epidemics remains unclear (particularly before large gatherings and festive holidays), community screening should be assessed in comparison to the costs of population level NPIs.

Another challenge for surveillance is its ability to assess the severity of an emerging variant, especially if the levels of both natural-acquired and vaccine-induced immunity are very different across countries.

## Protecting vulnerable groups and expanding behavioural research

Beyond spring 2022, there will likely remain population groups that are vulnerable to unfavourable outcomes, such as elderly people, people with underlying conditions, as well as immunocompromised and immunologically-naive people. In addition, because of the dynamically changing immunity landscape, immunity is also likely to wane for a larger share of the currently protected population during 2022. Although one can expect the protection to last longer against severe outcomes, individuals may underestimate their personal risk should their immunity have waned [14].

Two years into the pandemic, there is still insufficient understanding of the behavioural, cultural and societal drivers that either facilitate or inhibit population acceptance and adherence to public health interventions or on effective risk communication of these issues and how and why these drivers can change over time. To this end, a range of specific issues needs to be investigated using behavioural insights research methods. These methods include providing in-depth and actionable understanding about (i) how to continue to effectively promote COVID-19 vaccination acceptance and uptake rates (both for the primary vaccination course and additional booster doses), in particular in populations where there is active resistance to vaccination [15] and, (ii) within a context of pandemic fatigue [16], how to facilitate population acceptance of and adherence to any future NPIs should these become epidemiologically necessary. In relation to both these areas, it will also be necessary to systematically monitor and respond to misinformation that can reduce people’s willingness and motivation to undertake the respective public health measures [17]. To accomplish these goals, strengthening of behavioural insights research capacity is needed in many countries [18].

## Ensuring ‘vaccine agility’

As new variants of concern may emerge at unpredictable times and with unpredictable characteristics, these variants could affect the effectiveness of the immune response to current vaccine formulations. However, cell-mediated immunity from current vaccines, which is important for protection against severe disease, appears to show relatively good cross-protection against different variants [19].

Efficiency improvements are required for the full vaccine development cycle, from processes for selecting updated vaccine targets through to manufacturing. This might be based on lessons learned from a seasonal influenza model and should be based on a governance mechanism for strain selection. It should also include consideration of alternative vaccine strategies such as developing vaccines targeting conserved SARS-CoV-2 antigens that may offer broader protection against future variants or vaccines inducing strong mucosal immunity [20] and consideration of alternative vaccine strategies such as developing vaccines targeting conserved SARS-CoV-2 antigens that may offer broader protection against future variants or vaccines inducing strong mucosal immunity [20,21].

Furthermore, future efforts need to consider the speed at which vaccines can be distributed and administered to citizens at scale. This will require substantial global investments to develop and sustain such capacities, with resources to additionally monitor vaccine acceptance and reach the most vulnerable and at-risk population groups [15].

It is important to be clear about the objectives of future revaccinations of the general population and of vulnerable groups. Long-term vaccination strategies should align with public health priorities for managing COVID-19 burden as the situation (and population immunity) evolves, addressing immunity gaps and protecting the most at-risk populations. Depending on the objectives and the priorities as well as on the actual added benefit that can be obtained from additional doses, different needs for COVID-19 vaccine development and deployment may arise [22].

## Strengthening global health

The SARS-CoV-2 Omicron variant has intensified existing discussions surrounding global health equity and has demonstrated yet again that no country is safe until all countries are safe. Strategic decisions are required around enhanced investments in global health infrastructure and in bolstering availability of COVID-19 vaccines globally. In addition, there is a need to support all countries to strengthen their pandemic preparedness and to develop the capacity to conduct epidemiologic investigations [23]. Such investments would pay dividends beyond COVID-19.

Meanwhile, better understanding of possible pathways for viral evolution is needed. During the COVID-19 pandemic, there have been instances of SARS-CoV-2 transmission among a range of mammalian species, including mink in Europe or deer in North America [24], and there is a risk that the novel variants of concern (VOC) could emerge through zoonotic events. Therefore, greater emphasis on One Health aspects should be given at the global level.

## Addressing knowledge gaps

It is essential to recognise the many uncertainties that currently exist around factors that could have profound impact on the trajectory of the COVID-19 pandemic. Some uncertainties are intrinsic to the evolving epidemiological situation and are inherently unpredictable, such as the emergence of new variants or the negative impact on quality of life and economic productivity potentially arising from post-acute COVID. In addition, and in spite of the impressive scientific research effort that continues to be directed at COVID-19, there remains a lack of detailed understanding on key clinical aspects including susceptibility, mechanisms and duration of immunity, predictors of severity and correlates of protection. The multifaceted public heath response and increasingly divergent immunological status following multiple rounds of natural- and vaccine-induced exposure also makes assessment of the relative effectiveness of specific interventions ever more challenging. All of these factors are in principle amendable to research study, and there is a need for further and sustained investment and innovation to inform current and future public health policy.

## Conclusion

In the future, a new pandemic will occur, whether due to a very different new variant of SARS-CoV-2 or another pathogen altogether. Until then, it is essential to take advantage of the upcoming post-acute phase of the pandemic to foster recovery by identifying lessons from the pandemic and using these to strengthen pandemic preparedness and to design public health systems to efficiently manage COVID-19 over the long-term.

There are multiple uncertainties and conflicting factors currently at play. While individual and societal fatigue cannot be ignored, there is also the need for continued vigilance to the threats posed by COVID-19. Public health decisionmakers will need to be attentive to this dynamic while advocating for further public health work to reduce scientific uncertainties and to minimise the overall societal burden of COVID-19. It is also imperative to help citizens and communities to recover from the pandemic and to build societal resilience for future pandemics.
